# Peanut phytobezoar: an unusual cause for small bowel obstruction

**DOI:** 10.1093/jscr/rjae564

**Published:** 2024-08-28

**Authors:** Andrew J Sealey, Ngee-Soon Lau

**Affiliations:** General Surgery, Canberra Health Services, Yamba Drive, Garran 2605, Australia; General Surgery, Canberra Health Services, Yamba Drive, Garran 2605, Australia

**Keywords:** small bowel obstruction (SBO), bezoar, phytobezoar

## Abstract

Bezoars are indigestible masses of material forming within the gastrointestinal system. Phytobezoars are the most common subtype consisting of plant matter such as fibre, skins, and seeds. Rarely they present causing small bowel obstruction (SBO) and may be difficult to distinguish from faecalization on imaging. Here we present the case of a man in his 70s who rapidly consumed an expiring bag of peanuts and subsequently developed a SBO due to formation of a peanut phytobezoar. After failing conservative management, he required emergency surgery with intentional enterotomy to milk out the bezoar. This case highlights the importance of maintaining a broad differential and thorough history taking in patients presenting with SBO.

## Introduction

Small bowel obstruction (SBO) is a common emergency surgery presentation. Approximately 15% of patients admitted from emergency after presenting with abdominal pain are diagnosed with SBO [1]. The majority are caused by adhesions from prior surgery. Other mechanical causes for obstruction can be categorized as extrinsic including adhesions and hernia, intrinsic including malignancies, strictures and intussusception, and intraluminal obstructions as occurs in gallstone ileus, foreign body ingestion and bezoars.

Bezoars are a mass of undigested material within the gastrointestinal system formed from vegetable matter and seeds (phytobezoars), hair (trichobezoar), undigested milk (lactobezoar), and medications (pharmacobezoar). Complications of a bezoar may include bleeding, ulceration, perforation, intussusception, pancreatitis, obstructive jaundice, and SBO. They are a rare cause for SBO, accounting for between 0.4% and 4% of cases [2]. Risk factors include prior gastric surgery, impaired gastrointestinal motility, stenosis and stricturing, hypothyroid, abnormal chewing, and ingestion of a high fibre diet [3]. Here we present a rare case of a peanut phytobezoar presenting as SBO.

## Case presentation

A man in his 70s presented with a one-day history of abdominal pain and vomiting. He had not opened his bowels or passed flatus for one day prior to presentation. Recently he had consumed an entire large packet of peanuts as it was due to expire. His medical background was significant for a laparoscopic converted to open cholecystectomy for significant adhesions complicated by bleeding, requiring massive transfusion, and two returns to theatre. On initial review he was haemodynamically normal. His abdomen was distended and tender in the epigastrium without any features of peritonism. His haemoglobin was 156 g/L, white cell count 9.3x10^9^/L and C-reactive protein 0.4, liver function tests, urea, electrolytes, and creatinine were within reference ranges.

**Figure 1:**
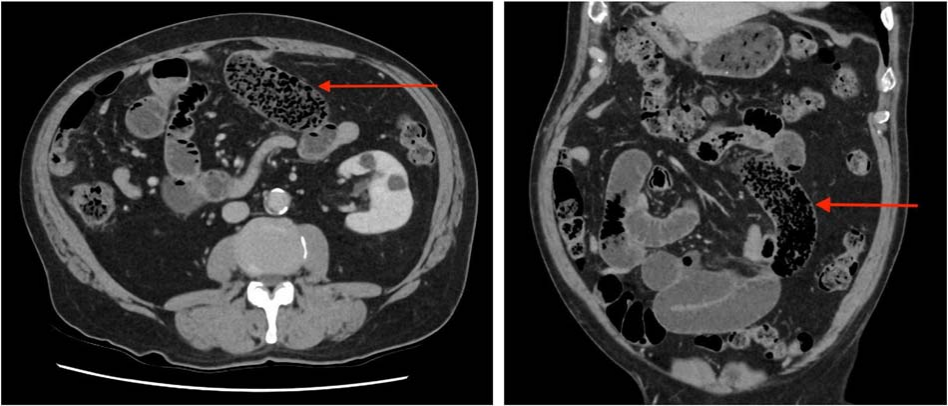
Abdominal CT scan axial (left) and coronal (right) images demonstrating ‘faecalization’ (arrow) of small bowel content.

A bowel obstruction was suspected, the patient was resuscitated with intravenous fluids, a nasogastric tube was inserted, and a computed tomography (CT) with intravenous (IV) contrast performed. On CT, multiple dilated loops of small bowel were identified with a transition point in the mid-abdomen (Fig. 1). The radiologist reported faecalization of small bowel contents at the transition which was thought to be consistent with an adhesional small bowel obstruction. The patient was admitted for a trial of conservative management. In the next 48 hours, he had failed to resolve and developed increasing pain and evolving peritonitis.

The patient was taken to theatres for emergency surgery. A laparoscopy was performed initially, which identified an SBO with a transition point in the mid-abdomen, centred on an adhesion to the anterior abdominal wall. The bowel was haemorrhagic, dusky and there was a palpable mass adjacent. The umbilical incision was extended to a mini-laparotomy and the small bowel delivered. A firm bezoar was easily palpated within the segment. A longitudinal enterotomy was made in healthy bowel and ~500 ml of undigested peanuts delivered (Video). The dusky segment of bowel improved and was not resected, the enterotomy was closed transversely in the Heineke–Mikulicz fashion. Post-operative progress was slow due to ileus; however, the patient recovered well and was discharged on post-operative day 8.

## Discussion

Bezoars are conglomerates of indigestible material forming within the gastrointestinal system. They are classified according to their composition, the four major types are phytobezoars, trichobezoars, pharmacobezoars, and lactobezoars. Phytobezoars are the most common, usually formed by seeds and plant matter from fruits and vegetables. In the case of our patient, he had no predisposing gastric motility disorders, but did rapidly consume a large volume of peanuts. Concretion typically occurs in the stomach where a bezoar may be asymptomatic, or cause pain, bleeding – due to pressure necrosis, nausea, vomiting, or anorexia [4]. A phytobezoar may pass into, or form de novo in the small bowel, in the latter presenting as SBO with transition point typically in the distal ileum where there is reduced small bowel diameter and motility and rarely they are associated with perforation [5]. In our patient, adhesions to the anterior abdominal wall may have formed a point of narrowing, facilitating the conglomeration of peanuts with subsequent obstruction.

Consumption of various organic foods is associated with development of phytobezoars. Worldwide, the most common is due to ingestion of persimmons, typically occurring in China, Japan and Mediterranean countries where their consumption is higher [6]. Other fruits, vegetables and seeds frequently associated with formation of phytobezoar are celery, pumpkin, grape peels, prunes, oranges, coconut, sunflower seeds, watermelon seeds, and mushrooms [4, 7]. Peanut bezoars are exceedingly rare, with only two known cases in the literature, both presenting with obstruction [8, 9]. One systematic review of case reports and series on seed phytobezoar over a 38 year period identified only one case caused by peanuts [10]. Our case therefore represents only the third published case of peanut phytobezoar.

Clinical features of SBO due to bezoar are indistinct from other causes such as adhesions. Most patients presenting acutely with symptoms of SBO will have a CT scan as they are rapid, non-invasive, and readily available. CT scans facilitate diagnosis and identification of underlying cause, and complications such as strangulation or perforation. Features of SBO on CT are dilated loops of small bowel proximally with collapsed loops distally, in 82% of cases there will be faecalization of bowel content [11]. On CT, faecalization is recognized as solid appearing content, typically distal within the small bowel, intermixed with air near the site of obstruction, in our patient both were identified. Interestingly, these features are similar to those observed with bezoar. A bezoar is oval or round, of soft-tissue density and also filled with air, found at the transition point [12]. Features that may favour a bezoar are a well-defined shape, an encapsulating wall, and a lesion in the stomach similar to the obstructing mass [12]. Unsurprisingly, diagnostic accuracy of bezoar is limited and misdiagnosis common [6]. Regarding our case, in retrospect our patients’ typical findings of faecalization were the bezoar. The similarities in these findings highlight the importance a thorough history and careful assessment of imaging where a bezoar is suspected, especially as these patients typically require surgery.

Management of phytobezoar varies with location. A gastric phytobezoar can often be successfully managed with dissolution, with resolution in up to 91% of cases [13]. Where this fails most patients will undergo endoscopic fragmentation and removal. Phytobezoars in the small bowel are not amenable to chemical dissolution and are often too distal for endoscopic management. For these patients, surgery is the treatment of choice. At surgery, attempts are made to fragment the bezoar and milk it to the caecum where it should not re-obstruct [14]. If extra-intestinal fragmentation is not feasible, an enterotomy should be performed in healthy bowel to extract the bezoar as was performed in our patient. The traditional approach to surgery for a SBO in a non-virgin abdomen is laparotomy, however, with improvements in laparoscopic expertise the laparoscopic approach is preferred by some surgeons for select patients. A retrospective study comparing a conventional open to laparoscopic approach for SBO due to bezoar specifically found the laparoscopic approach +/− mini laparotomy to be safe, effective, and associated with a shorter length of stay without increased risk of complication [15]. In our case, the patient failed an initial trial of conservative management and required an emergency operation. An initial laparoscopic approach was used to facilitate assessment and adhesiolysis. Due to firmness on palpation and the condition of the involved bowel segment, extra-intestinal fragmentation was not attempted. Instead, a mini-laparotomy was performed to safely perform an enterotomy and extract the bezoar.

## Conclusion

Intestinal obstruction due to bezoar is a rare but important phenomenon that may be difficult to identify on imaging. Rapid ingestion of large volumes of organic matter in patients presenting with SBO should raise suspicion. To the best of our knowledge, this is only the third published case of SBO due to a peanut phytobezoar.

## Supplementary Material

peanut_bezoar_rjae564
